# Hypoxically cultured cells of oral squamous cell carcinoma increased their glucose metabolic activity under normoxic conditions

**DOI:** 10.1371/journal.pone.0254966

**Published:** 2021-10-22

**Authors:** Yuta Shinohara, Jumpei Washio, Yuri Kobayashi, Yuki Abiko, Keiichi Sasaki, Nobuhiro Takahashi

**Affiliations:** 1 Division of Oral Ecology and Biochemistry, Tohoku University Graduate School of Dentistry, Sendai, Japan; 2 Division of Advanced Prosthetic Dentistry, Tohoku University Graduate School of Dentistry, Sendai, Japan; Instituto Nacional de Cardiologia, MEXICO

## Abstract

**Objective:**

The oxygen concentration within cancer tissue is known to be low, but is expected to increase rapidly when oxygen is supplied by angiogenesis and hematogenous metastasis, suggesting that rapid increases in oxygen levels might influence cancer cell physiology. Therefore, we investigated the effects of oxygen concentration fluctuations on the glucose metabolism of cancer cells.

**Methods:**

The glucose metabolism of oral squamous cell carcinoma (HSC-2 and HSC-3) and normal epithelial (HaCaT) cells cultured under normoxic (21% oxygen) or hypoxic (1% oxygen) conditions was measured using a pH-stat system under normoxic or hypoxic conditions. The acidic end-products and reactive oxygen species (ROS) generated by glucose metabolism were also measured.

**Results:**

Under normoxic conditions, the metabolic activity of hypoxically cultured cancer cells was significantly increased, and the production of acids other than lactate was upregulated, while the normal cells did not respond to rapid increases in oxygen levels. ROS production was higher in normoxic conditions in all cells, especially the hypoxically cultured HSC-3 cells.

**Conclusions:**

Rapid increases in oxygen levels might enhance the glucose metabolism of hypoxically cultured cancer cells by mainly activating the TCA cycle and electron transport system, which might activate cancer cells through the ATP and ROS generation.

## Introduction

Every year, about 350,000 people are newly diagnosed with head and neck cancer, especially oral cancer [1]. Generally, oral cancer is treated using surgery or radiotherapy. These treatments often result in the loss of oral functions (eating, speaking, etc.), esthetic disorders, and/or decreased salivary secretion and can have a significant impact on patients’ quality of life (QOL). Therefore, early diagnosis and minimal treatment are desired. For this purpose, basic research into cancer tissues and cells has been widely performed. Since the 1980s, various oncogenes [2] and tumor suppressor genes [3] have been discovered. In addition, these genes have been found to regulate cell metabolism [4]; hence, the metabolic alterations caused by mutations have attracted attention as a common feature of cancer cells. In particular, energy production-related metabolic pathways, which support the infinite and rapid growth of cancer cells, are essential components of cancer biology [5].

Mammalian cells mainly produce ATP via oxidative phosphorylation under normoxic conditions. However, cancer cells increase their uptake of glucose and glycolysis and subsequently produce energy via a pathway that produces lactate, even in the presence of sufficient oxygen [6]. This phenomenon is called the “Warburg effect” and is a well-known metabolic characteristic of cancer cells [7]. ^18^F-fluorodeoxyglucose (FDG) positron emission tomography (PET), an imaging modality used for clinical cancer diagnosis, is based on this phenomenon. Recently, however, it has been shown that ATP is also produced in tumor cells under aerobic conditions through oxidative phosphorylation by mitochondria [8–10].

The environment surrounding cancer tissue is unique and is known to be hypoxic [11]. In rapidly growing cancer tissue, oxygen consumption is increased, oxygen supply is decreased due to the abnormal blood vessel structures found in such tissue, the oxygen diffusion distance is increased, and blood flow is temporarily blocked, resulting in the development of a hypoxic region [12]. Thus, the presence of a hypoxic region is regarded as an important indicator for clinically diagnosing cancer [13]. PET imaging using ^18^F-fluoromisonidazole (FMISO), which is specifically taken up into hypoxic tissue, has been applied to the diagnosis of various tumors, including oral cancer [14]. Therefore, increasing numbers of studies on cancer physiology are being performed under hypoxic conditions in order to reveal the physiological and malignant characteristics specific for cancer cells [8, 15, 16]. Moreover, the partial pressure of oxygen varies within the same cancer tissue, and an oxygen concentration gradient from the periphery to the interior of cancer tissue has been observed on FMISO-based PET imaging [14, 17]. It is also assumed that the partial pressure of oxygen in cancer tissue increases rapidly when blood flow increases due to angiogenesis or the dissemination of cancer cells by blood flow. These observation and speculation suggest that cancer cells are exposed to dynamic changes in oxygen partial pressure and might modulate their physiological and malignant properties accordingly. Therefore, metabolic studies involving not only hypoxic environments, but also fluctuations in oxygen concentrations, are necessary.

In this study, we evaluated the effects of fluctuations in the environmental oxygen concentration on the metabolism of glucose in cancer cells using a real-time cell metabolism monitoring system [18]. Cancer cells were cultured under normoxic (21% oxygen in air) or hypoxic conditions (1% oxygen), and then the metabolic activity of these normoxically or hypoxically cultured cells was evaluated under normoxic or hypoxic conditions. We also tried to examine the metabolic regulatory mechanisms in operation in such cells by measuring the production of lactate and reactive oxygen species (ROS), which are closely related to metabolic pathways.

## Materials and methods

### Cell lines

Human squamous carcinoma cell-derived strains; i.e., HSC-2 (JCRB0622: oral squamous carcinoma cells derived from weak gingivae) and HSC-3 (JCRB0623: oral squamous carcinoma cells derived from highly advanced tongue cancer) (JCRB Cell Bank, Tokyo, Japan) cells, and a normal human keratinized epithelial cell line, HaCaT (Cosmo Bio Co., Ltd., Tokyo, Japan), were used. All cell lines were authenticated by means of short-tandem repeat (STR) analysis.

### Cell culture and evaluation of cell proliferation

Each cell type was cultured as recommended. The cancer cells; i.e., the HSC-2 and HSC-3 cells, were cultured in Eagle’s minimal essential medium (Wako Pure Chemical Industries Ltd., Tokyo, Japan) supplemented with 2 mmol/L L-alanyl-L-glutamine solution (Wako Pure Chemical Industries Ltd., Tokyo, Japan), 10% heat-inactivated fetal bovine serum, 100 μg/mL streptomycin, and 100 U/mL penicillin, while the HaCaT cells were cultured in Dulbecco’s modified Eagle’s medium (10-013-CVR; Corning, NY, USA) supplemented with 10% heat-inactivated fetal bovine serum, 100 mg/mL streptomycin, and 100 U/mL penicillin. The normoxic culture (oxygen concentration: 21%) was performed at 37°C with 5% humidified CO_2_ (MCO-18AC CO_2_ incubator; Sanyo, Tokyo, Japan), while the hypoxic culture (oxygen concentration: 1%) was performed at 37°C with 5% humidified CO_2_ (CPO2-2301 O_2_/CO_2_ incubator; Hirasawa, Tokyo, Japan). All the cells were subcultured to maintain logarithmic growth. The cells were detached from their dishes and collected by adding trypsin for 5 min, on day 3 for cancer cells or on day 5 for normal cells before being cultured at 80–90% confluence. They were then suspended in saline at a density of 5.0 × 10^6^ cells/mL, which was assessed using a cell counter (Countess II FL; Thermo Fisher Scientific, Waltham, Massachusetts, USA). The cells grown in hypoxic conditions were temporarily centrifuged in a normoxic environment at 4°C (for 5 min) and quickly returned to hypoxic conditions. These cell suspensions (3-day cultured cancer cells and 5-day cultured normal cells) were used for all the following experiments.

For the evaluation of cell proliferation, the cell suspension was transferred to new culture dishes and incubated under normoxic or hypoxic conditions, as described above. The number of cells was periodically determined using a cell counter, as described above.

### Evaluation of glucose metabolism activity under normoxic and hypoxic conditions

Metabolic activity was evaluated using pH-stat systems [18] in both the normoxic (21% oxygen in air) and hypoxic environments (1% oxygen) (for normoxic experiments: AUT-211S AUTO pH-stat system; TOA Electronics, Aichi, Japan; for hypoxic experiments: AT-710M pH-stat system; Kyoto Electronics Industry, Kyoto, Japan). These systems can estimate metabolic activity based on acid production (lactic acid, CO_2_-derived carbonic acid, etc.) from glucose. The pH-stat system used for the hypoxic experiments was placed in the hypoxic chamber, in which hypoxic conditions were created by diluting and replacing the gas in the anaerobic glove chamber (ANB-180-P; Hirasawa, Tokyo, Japan) with nitrogen gas. The reaction mixture contained 1 mL of the cell suspension (5 × 10^6^ cells/mL) and 3.75 mL of physiological saline and was pre-incubated at 37°C for 10 min. Then, 0.25 mL of 100 mM glucose was added to the mixture (final concentration: 10 mM), and acid production was monitored for 20 min using the pH-stat system. The reaction mixture was then immediately centrifuged at 1000 rpm and 2°C for 5 min, and the resultant supernatant was stored at -80°C until it was used for the analysis of acidic end-products. As a control, the reaction mixture without glucose was also treated and stored in the same way. Before and after the incubation procedure, the viability of the cells was confirmed using 0.4% (w/v) trypan blue solution (Wako Pure Chemical Industries Ltd., Tokyo, Japan).

### Analysis of acidic end-product levels

After being thawed, the stored supernatants were centrifuged again (at 1,000 g and 5°C for 7 min), and the resultant supernatants were filtered through a polypropylene membrane (pore size: 0.20 μm; Toyo Roshi Ltd., Tokyo, Japan). The sample was analyzed by HPLC (high-performance liquid chromatography) (Shimadzu Prominence LC-20AD; Shimadzu Corporation, Kyoto, Japan) to determine the levels of acetic acid, lactic acid, formic acid, malic acid, fumaric acid, succinic acid, citric acid, α-ketoglutaric acid, oxalic acetic acid, and pyruvic acid.

### The effect of rotenone on the glucose metabolic activity and lactate production

The effect of 5 μM rotenone (MP biomedicals, Santa Ana, USA) on the glucose metabolism of HSC-3 cells cultured hypoxically was evaluated using pH-Stat system and HPLC, as described above. The concentrations of rotenone was set based on previous reports [19, 20].

### Production of ROS during glucose metabolism

The amount of ROS produced during glucose metabolism was measured using the Cell Meter^™^ fluorescent intracellular ROS activity assay kit (Amplite^™^ ROS deep red; AAT Bioquest, Inc., Sunnyvale, CA, USA). This reagent can detect the hydrogen peroxide, hydroxyl radicals, and tert-butyl hydroperoxide produced in cells. Before the monitoring of metabolic activity, Amplite^™^ ROS deep red was added to the cell suspension, which was then pre-incubated at 37°C for 20 min in normoxic or hypoxic conditions. Subsequently, glucose metabolism activity was monitored using a pH-stat system, as described above. The reaction mixture was collected before and after glucose metabolism, before being added to a 96-well plate, and fluorescence intensity (excitation wavelength: 658 nm; measurement wavelength: 675 nm) was immediately measured using a fluorescence microplate reader (Varioskan Flash; Thermo Fisher Scientific, Waltham, Massachusetts, USA).

### Statistical analysis

The t-test was used for comparisons between two groups. In all cases, p-values of <0.05 were considered significant. The data were analyzed using StatFlex Ver. 6 (Artech Co., Ltd., Osaka).

## Results

### Effects of the environmental oxygen concentration on cell growth

**Fig 1:**
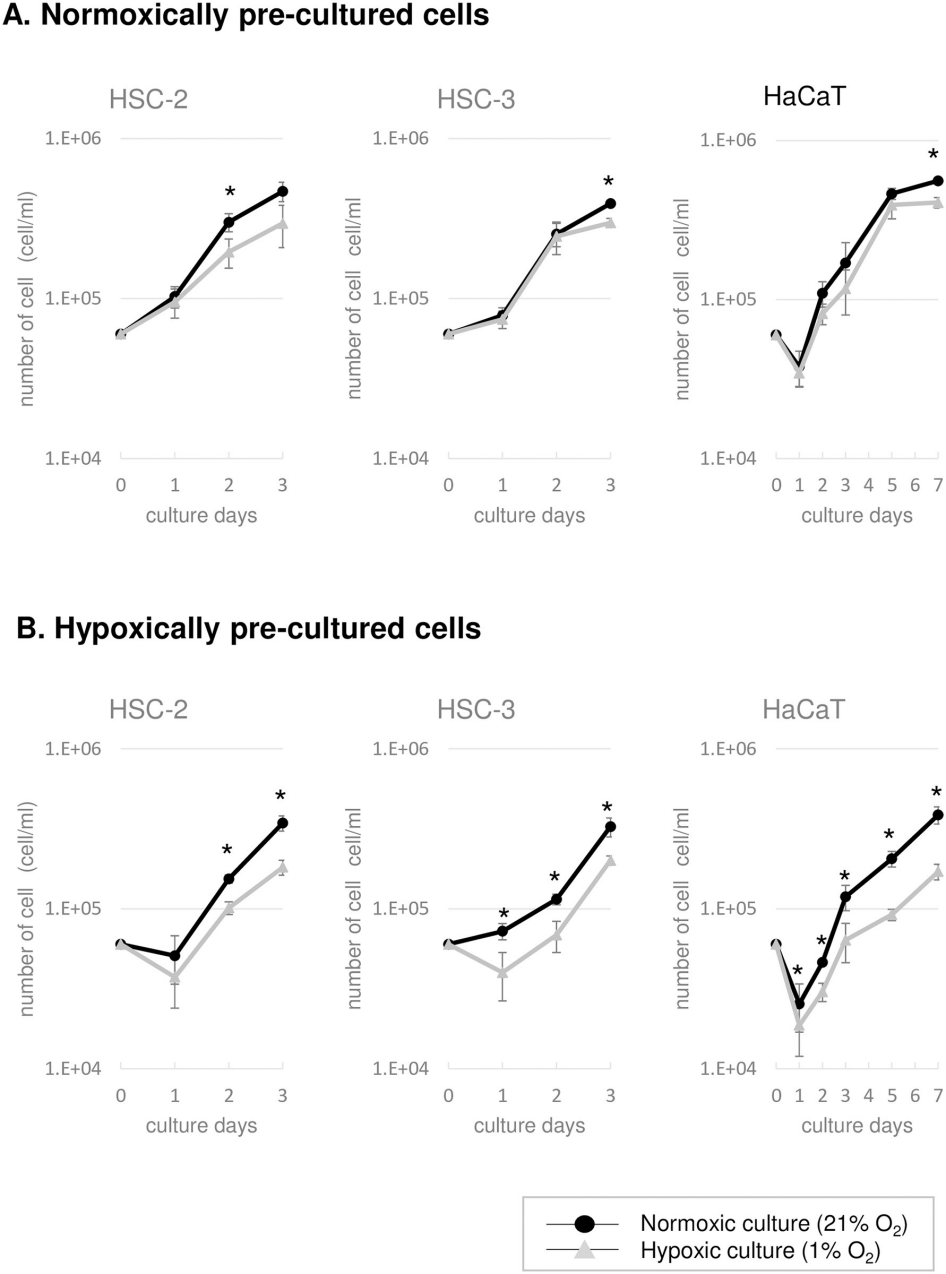
Cell growth in normoxic and hypoxic conditions. Cells that had been pre-cultured in normoxic (A) or hypoxic (B) conditions were cultured under normoxic or hypoxic conditions. The error bars represent standard deviation. *Significant difference in the number of cells (p <0.05) between the normoxic and hypoxic culture conditions, according to the paired t-test.

All of the cells were basically able to proliferate in both normoxic and hypoxic conditions (Fig 1); however, the cells that were pre-cultured in hypoxic conditions exhibited a longer lag phase than those that were pre-cultured in normoxic conditions, especially when the cells were cultured under hypoxic conditions. Once the cells started to proliferate, they exhibited similar growth rates in both normoxic and hypoxic conditions.

### Effects of the environmental oxygen concentration on glucose metabolism

**Fig 2:**
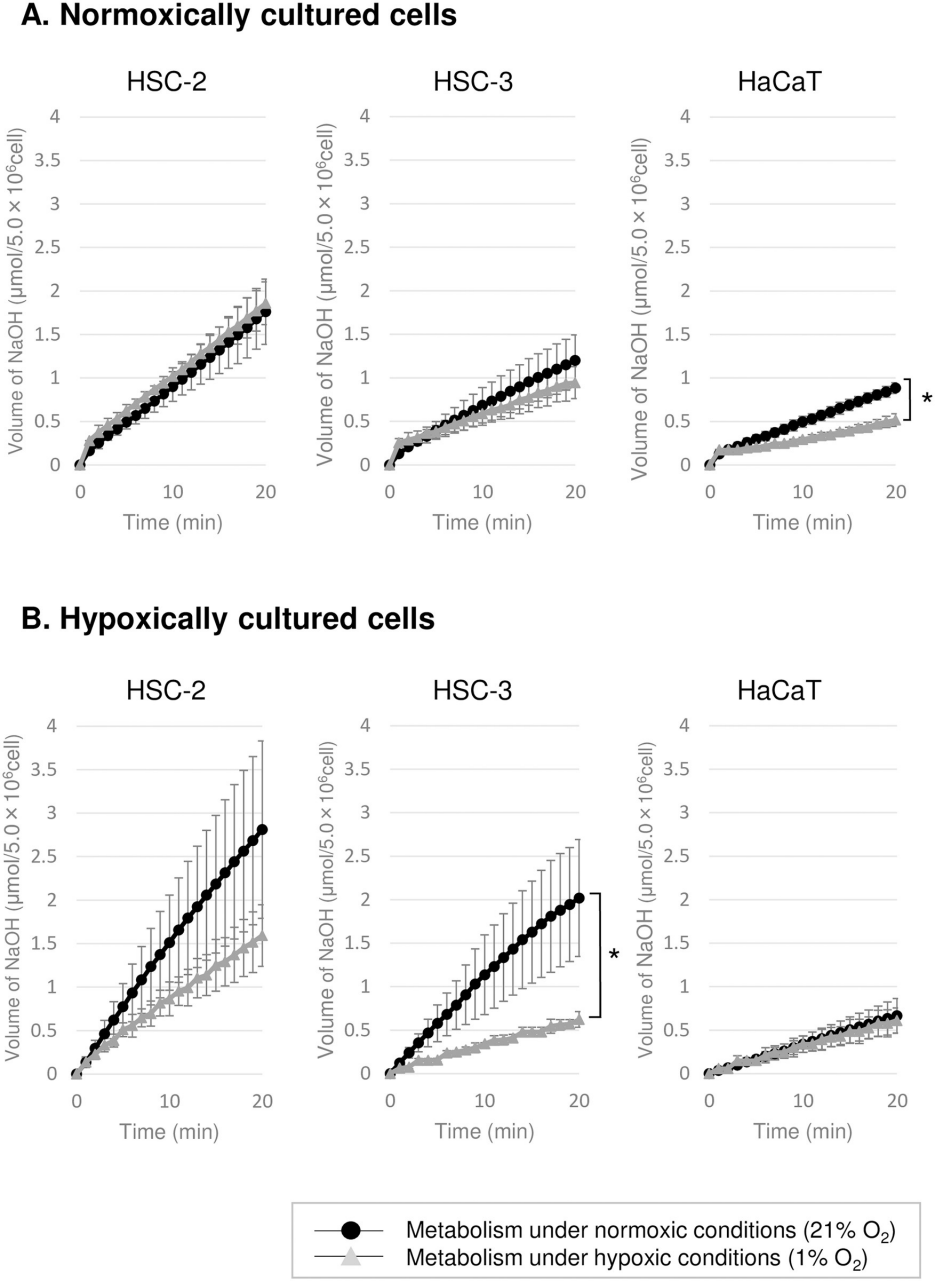
Glucose-derived acid production. Glucose-derived acid production by (A) normoxically and (B) hypoxically cultured cells was measured as the amount of NaOH added by the pH-stat system under normoxic or hypoxic conditions. Cancer cells were harvested after 3-day culture and normal cells were harvested after 5-day culture. The error bars represent standard deviation. Significant difference (*, p <0.05) between the normoxic and hypoxic conditions, according to the paired t-test.

No acid production was observed before the addition of glucose, and only after the addition of glucose the acid production was observed, indicating that the acid production was derived from glucose metabolism (data not shown). No significant differences in the glucose metabolism activity of the cancer cells (HSC-2 and HSC-3) cultured in normoxic conditions were detected between normoxic and hypoxic conditions. However, the normal (HaCaT) cells displayed significantly lower metabolic activity under hypoxic conditions (0.54 to 0.63 times, p <0.05) (Fig 2). On the other hand, the hypoxically cultured cancer cells demonstrated higher metabolic activity under normoxic conditions (2.02 to 4.79 times, p <0.05, in HSC-3 cells; 1.44 to 2.03 times, p = 0.06, in HSC-2 cells). The metabolic activity of the hypoxically cultured HaCaT cells was not influenced by normoxic conditions.

### Effects of the environmental oxygen concentration on the production of acidic end-products

**Fig 3:**
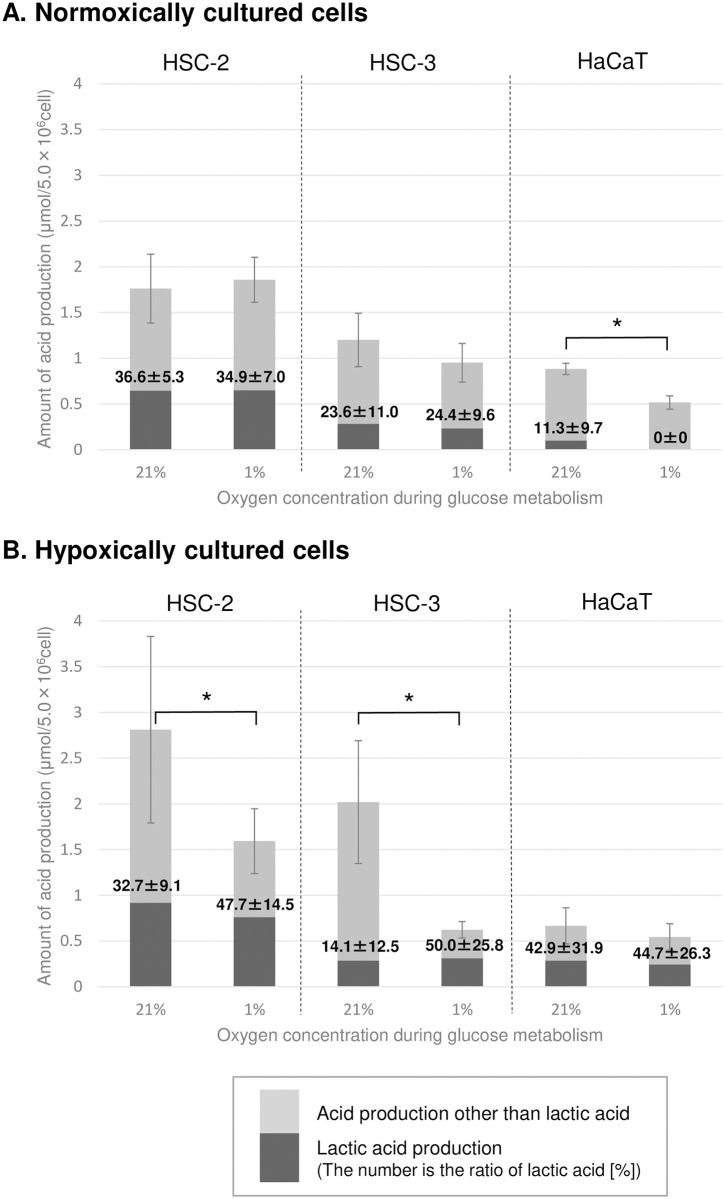
Production of lactic acid and other acids produced during glucose metabolism. The amount of other acids produced by (A) normoxically and (B) hypoxically cultured cells was calculated by subtracting the amount of lactic acid produced from total acid production, which was obtained from Fig 1. The error bars represent standard deviation. Significant difference (*, p <0.05) in the amount of acid other than lactic acid between normoxic and hypoxic conditions, according to the paired t-test.

Lactic acid was the only acidic end-product detected with HPLC analysis. Acetic acid, formic acid, malic acid, fumaric acid, succinic acid, citric acid, α-ketoglutarate, oxalic acid, and pyruvate were not detected. Therefore, the acids produced other than lactic acid were assumed to be carbonic acid derived mainly from the CO_2_ produced by pyruvate dehydrogenation and the TCA cycle in glucose metabolism. Then, the ratio of the amount of lactic acid, as measured by HPLC, to the total amount of acids, as measured by a pH-stat system (Fig 2), was calculated (Fig 3).

The normoxically cultured HSC-2 and HSC-3 cells exhibited high lactic acid ratios (23.6–36.6%), and the lactic acid ratios of these cells were not affected by the environmental oxygen concentration. The lactic acid ratio of the normoxically cultured HaCaT cells was <11.3%. On the other hand, the hypoxically cultured HSC-2 and HSC-3 cells displayed markedly increased total acid production under normoxic conditions; i.e., significant increases in their levels of acids other than lactic acid were seen (HSC-2: 2.31±0.89 times, p <0.05; HSC-3: 6.92±3.84 times, p <0.05). The hypoxically cultured HaCaT cells produced lactic acid regardless of the environmental oxygen concentration. Their lactic acid ratio was about 50%.

### The effect of rotenone on the glucose metabolic activity and lactate production

**Fig 4:**
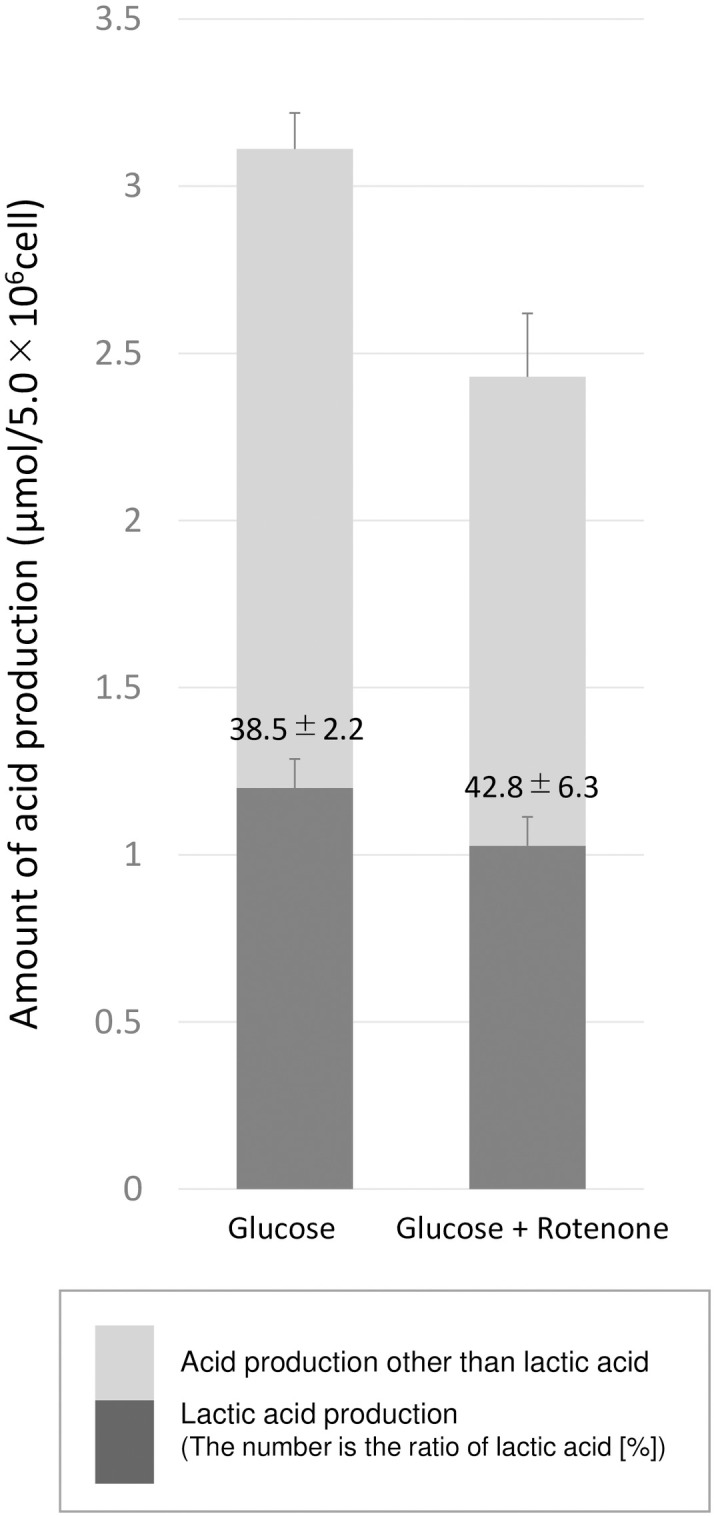
The effects of rotenone on the production of lactic acid and other acids during glucose metabolism by hypoxically cultured HSC-3. The amount of other acids produced by hypoxically cultured HSC-3 cells was calculated by subtracting the amount of lactic acid produced from total acid production, which was obtained with pH-stat system. The number is the ratio of lactic acid [%]. The error bars represent standard deviation.

Using hypoxically cultured HSC-3 cells that showed a greater increase in metabolic activity under the normoxic conditions, the effects of the addition of rotenone on their glucose metabolism activity (total acids and lactate production) were evaluated. Rotenone suppressed total acid production by 22% and lactic acid production by 14.5% compared with those under normoxic condition without rotenone (Fig 4).

### Effects of the environmental oxygen concentration on ROS production

**Fig 5:**
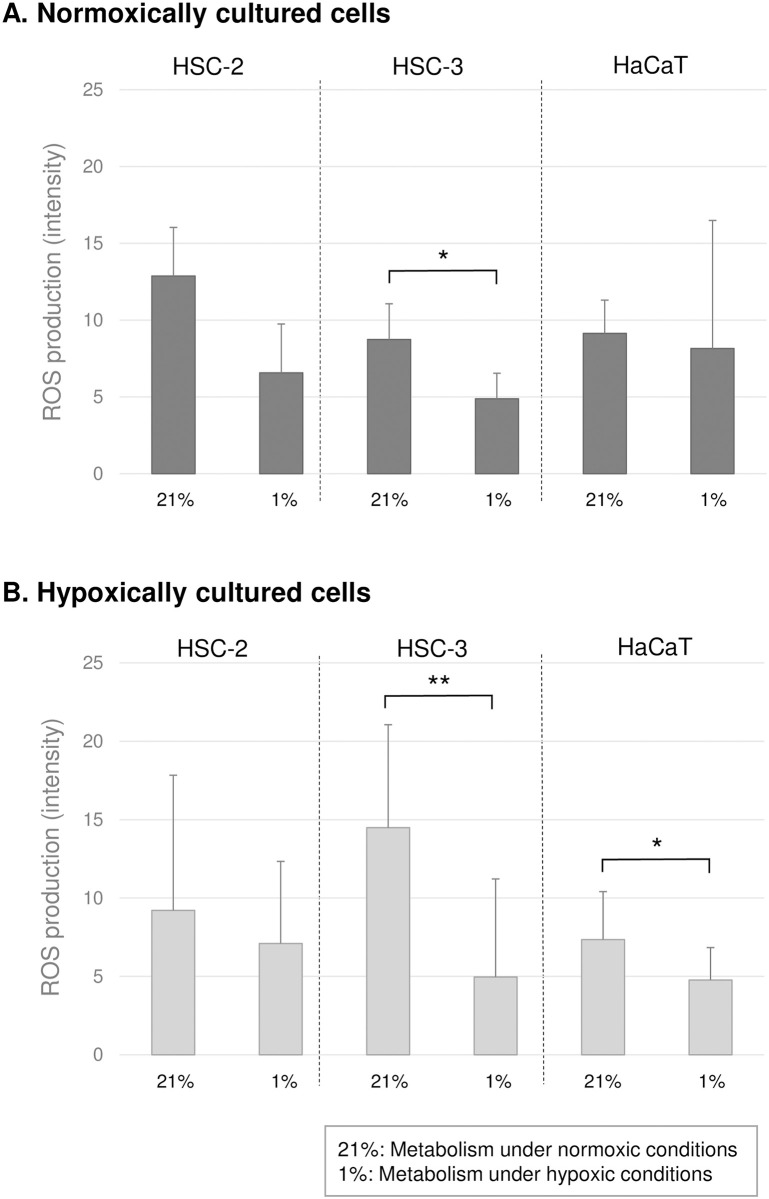
Production of Ros during glucose metabolism. The amount of ROS produced by (A) normoxically and (B) hypoxically cultured cells was estimated by ROS assay kit. Cancer cells were harvested after 3-day culture and normal cells were harvested after 5-day culture. The error bars represent standard deviation. Significant difference (*, p <0.05; **, p <0.001) in the amount of other acids produced between normoxic and hypoxic conditions, according to the paired t-test.

All the cells produced higher amounts of ROS during glucose metabolism in normoxic conditions, regardless of the culture conditions (Fig 5). In particular, the HSC-3 cells cultured in hypoxic conditions exhibited the greatest ROS production in normoxic conditions.

## Discussion

In terms of cell proliferation, no clear difference in the cell growth rate was detected between normoxic and hypoxic conditions in the present study although the lag phase of growth was prolonged particularly when cultured under hypoxic conditions (Fig 1), suggesting that both cancer and normal cells are able to grow under hypoxic conditions after these cells adapt to hypoxic conditions during the lag phase of growth. The cell physiology of cancer cells after adaptation to hypoxic conditions has been reported in the previous papers [8, 15, 16], in which the common conclusion is that glycolysis was enhanced for energy production while the contribution of oxidative phosphorylation was suppressed under hypoxic conditions. However, these data do not reveal the effect of rapid changes in oxygen concentration on the cell physiology. Therefore, we attempted to demonstrate this effect using the real-time cell metabolic activity monitoring system [18] together with a hypoxic chamber, and showed that rapid changes from normoxic to hypoxic conditions, or vice versa, influenced metabolic activity and caused metabolic shifts in both normal and cancer cells (Figs 2–5). These results were unexpected based on the cell proliferation data, but strongly suggest that sudden changes in the environmental oxygen concentration cause relatively rapid metabolic shifts, especially when hypoxically cultured cancer cells are exposed to rapid increases in oxygen levels, and the resultant metabolic modifications might induce cellular adaptive responses, which aim to promote survival in the new environment.

**Fig 6:**
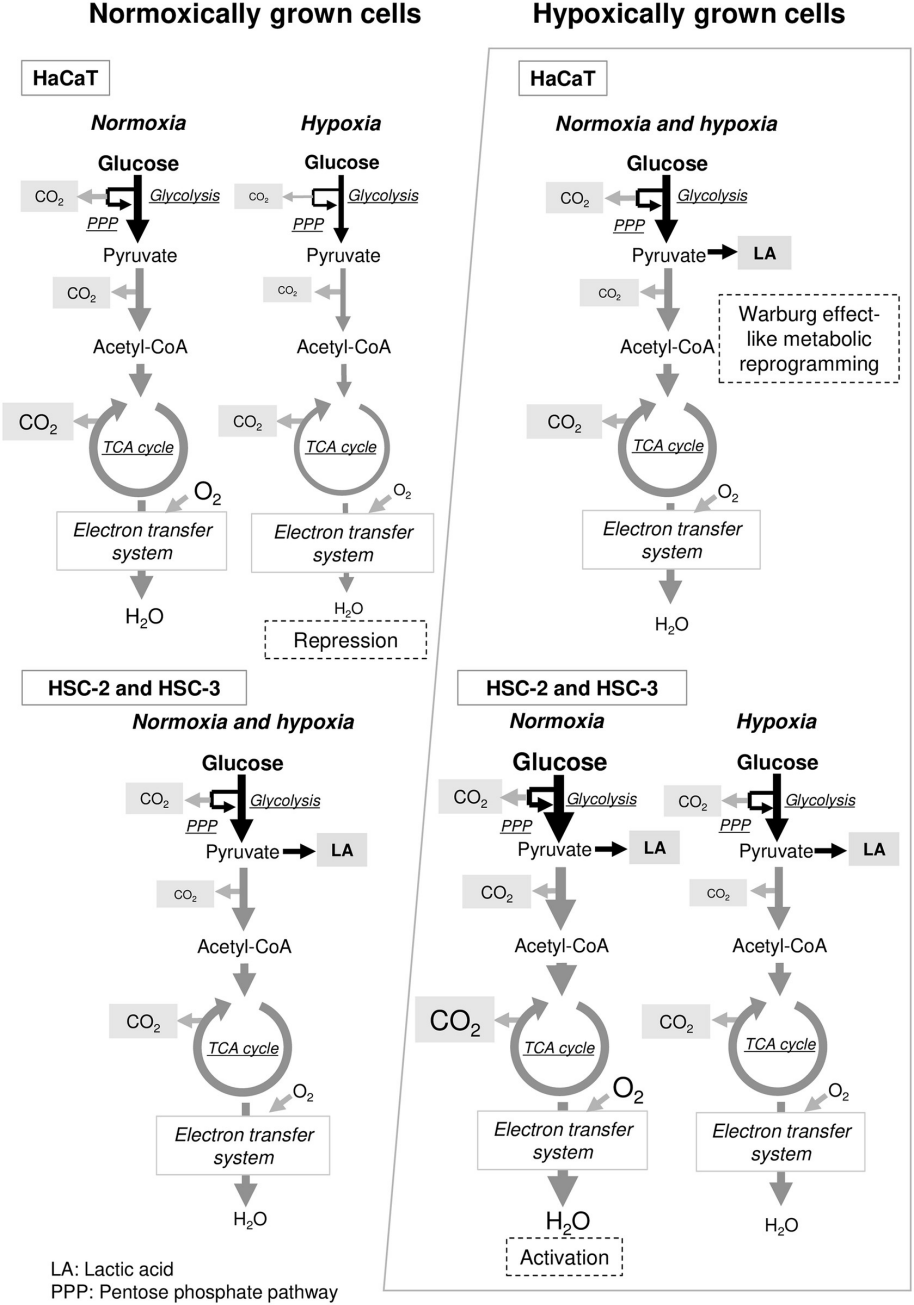
Schematic diagram showing the metabolic effects of the environmental oxygen concentration revealed in the present study.

In the normoxically cultured cancer cells, there was no significant difference in glucose metabolism activity between the normoxic and hypoxic conditions (Figs 2 and 3), indicating that cancer cells are able to continue to metabolize glucose even when the environmental oxygen level decreases rapidly. In other words, normoxically cultured cancer cells have the ability to metabolize glucose over a wide range of oxygen concentrations (at least 1–21%). In addition, these cells displayed similar levels of lactate production in both the normoxic and hypoxic conditions (Fig 3), indicating that cancer cells can perform glycolysis in normoxic conditions (known as the Warburg effect) (Fig 6). On the other hand, the normoxically cultured normal cells (HaCaT cells) exhibited significantly decreased glucose metabolism activity in hypoxic conditions (Fig 2), indicating that normal cells cannot response to rapid reductions in the environmental oxygen concentration (Fig 6). This could be due to their dependency on oxidative metabolic pathways for glucose metabolism, which is supported by the low levels of lactate production seen in these cells (Fig 3). However, the fact that similar proliferation rates were observed in both normoxic and hypoxic conditions (Fig 1) suggests that such metabolic repression is temporary and that normal cells might adapt to hypoxia during proliferation. Such adaptations might involve the induction/activation of oxidative metabolic enzymes that can function even in hypoxic conditions and/or alternative metabolic pathways involving other metabolic substrates, such as glutamine [21]. In the previous reports, it was suggested that not all lactate was produced by glucose metabolism, but also by glutamine metabolism (glutaminolysis) [8, 22–24]. However, in the experimental condition in the present study, the acid production was observed only after the addition of glucose into the reaction mixture with cells, which suggests that there was no lactate production by endogenous glutamine. Differences in cell type may be one of the factors that occur such differences.

In contrast, the glucose metabolism activity of the hypoxically cultured cancer cells increased dramatically under normoxic conditions (Fig 2), and this increase was accompanied by increased production of acids other than lactic acid (assumed to be carbonic acid derived from CO_2_) (Fig 3), suggesting that these cancer cells have the potential to activate glucose metabolism by upregulating oxidative metabolic pathways, such as the TCA cycle and electron transfer system, when oxygen is available (Fig 6). To validate this hypothesis, the effects of rotenone on cellular glucose metabolism were evaluated (Fig 4). The increased acid production (other than lactic acid) exhibited under normoxic condition by hypoxically cultured HSC-3 cells was suppressed by the addition of rotenone. Rotenone is known to inhibit the electron transfer of ubiquinone (coenzyme Q) in the electron transfer system. Therefore, the decrease in acid production (other than lactic acid) by rotenone may be due to the inhibition of the metabolic system of oxidative phosphorylation (from the TCA cycle to the electron transfer system), which in turn reduces acid production by the TCA cycle (presumably carbon dioxide). On the other hand, acid production (other than lactic acid) was observed even in the presence of rotenone (Fig 4), suggesting that the pentose phosphate pathway, a side pathway of the glycolysis, was functioning, and that carbon dioxide produced in the pentose phosphate pathway was measured as an acid other than lactic acid. This phenomenon that the increase in oxygen supply activates cellular glucose metabolism seems to be opposite to the "Pasteur effect" [25], in which the glucose consumption rate decreases and is accompanied by a shift in glucose metabolism from glycolysis to the TCA cycle and electron transport system when oxygen levels increase. A further study is required to elucidate the mechanism responsible for the abovementioned phenomenon. Furthermore, the cancer cells continued to produce lactate in the presence of rotenone (Fig 4). As discussed above, it is unlikely that these cancer cells produce lactate from glutamine under the experimental conditions without glutamine. Therefore, the lactate produced in the presence of rotenone was most likely produced from glucose by the glycolysis. This could be explained by the fact that glucose metabolism in cancer cells is highly dependent on the glycolysis (known as the Warburg effect).

In cancer tissues, it is considered that chronic hypoxia and the rapid recovery of the environmental oxygen concentration due to angiogenesis and cancer cell dissemination occur repeatedly [26]. In addition, it has been reported that rapid fluctuations in the environmental oxygen level lead to the activation of cancer cells. Rofstad found that when mice that had been seeded with human malignant melanoma cells were periodically exposed to a hypoxic environment (8%) (10 min x 12 times/day, for about 2 weeks), tumor metastasis was increased compared with that seen in the control group, which was not exposed to hypoxic conditions [27]. The activation of glucose metabolism due to a rapid increase in the environmental oxygen concentration caused by angiogenesis and/or cancer cell dissemination might increase energy production since oxidative phosphorylation-based ATP production is more efficient than glycolysis and enhances the proliferation of cancer cells. Furthermore, the upregulation of ROS production in response to normoxic conditions (Fig 5) might function as a redox signal that induces/activates metastatic potential [28] rather than cell impairment or apoptosis [29]. However, it seemed that the enhancement of metabolic activity observed in the hypoxically cultured cancer cells was transient because the growth rate of the cancer cells was similar in both normoxic and hypoxic conditions (Fig 1), and in the normoxic conditions the metabolic rate of the normoxically cultured cancer cells was not as high as that of the hypoxically cultured cells (Fig 2).

The hypoxically cultured normal cells displayed similar rates of glucose metabolism and lactate production under both normoxic and hypoxic conditions (Figs 2 and 3), suggesting that during hypoxic proliferation glucose metabolism is reprogrammed from a metabolic pathway that is mainly oxidative (the TCA cycle and electron transport system) to a metabolic pathway that is partly glycolytic, e.g., a phenomenon similar to the Warburg effect (Fig 6). Further study is needed to elucidate its molecular mechanisms.

## Conclusions

The present study clearly demonstrated that rapid changes in the environmental oxygen concentration affect cellular glucose metabolism. Interestingly, only the hypoxically cultured cancer cells exhibited enhanced glucose metabolism, along with a metabolic shift from glycolytic to oxidative pathways in response to a rapid increase in the environmental oxygen concentration. This finding suggests that a rapid increase in the environmental oxygen concentration activates cancer cells by increasing the ATP supply through oxidative phosphorylation and signaling modifications induced by ROS production. A metabolic shift might be the first adaptive response to a rapid change in the environmental oxygen concentration and cell properties might subsequently be modified through the expression of various genes.

## Supporting information

S1 Data (XLSX)

## References

[1] Bray F, Ferlay J, Soerjomataram I, Siegel RL, Torre LA, Jemal A. Global cancer statistics 2018: GLOBOCAN estimates of incidence and mortality worldwide for 36 cancers in 185 countries. CA Cancer J Clin. 2018;68:394–424. doi: 10.3322/caac.21492 30207593

[2] Gabay M, Li Y, Felsher DW. MYC activation is a hallmark of cancer initiation and maintenance. Cold Spring Harb Perspect Med. 2014;4. doi: 10.1101/cshperspect.a014241 24890832 PMC4031954

[3] Marcel V, Catez F, Diaz J-J. p53, a translational regulator: contribution to its tumour-suppressor activity. Oncogene. 2015;34:5513–5523. doi: 10.1038/onc.2015.25 25728674

[4] Cairns RA, Harris IS, Mak TW. Regulation of cancer cell metabolism. Nat Rev Cancer. 2011;11:85–95. doi: 10.1038/nrc2981 21258394

[5] Washio J, Takahashi N. Metabolomic studies of oral biofilm, oral cancer, and beyond. Int J Mol Sci. 2016;17. doi: 10.3390/ijms17060870 27271597 PMC4926404

[6] Warburg O. On the origin of cancer cells. Science. 1956;123:309–314. doi: 10.1126/science.123.3191.309 13298683

[7] Liberti MV, Locasale JW. The Warburg effect: How does it benefit cancer cells? Trends Biochem Sci. 2016;41:211–218. doi: 10.1016/j.tibs.2015.12.001 26778478 PMC4783224

[8] Pacheco-Velázquez SC, Robledo-Cadena DX, Hernández-Reséndiz I, Gallardo-Pérez JC, Moreno-Sánchez R, Rodríguez-Enríquez S. Energy metabolism drugs block triple negative breast metastatic cancer cell phenotype. Mol Pharm. 2018;15:2151–2164. doi: 10.1021/acs.molpharmaceut.8b00015 29746779

[9] Moreno-Sánchez R, Marín-Hernández A, Saavedra E, Pardo JP, Ralph SJ, Rodríguez-Enríquez S. Who controls the ATP supply in cancer cells? Biochemistry lessons to understand cancer energy metabolism. Int J Biochem Cell Biol. 2014; 50:10–23. doi: 10.1016/j.biocel.2014.01.025 24513530

[10] Rodríguez-Enríquez S, Hernández-Esquivel L, Marín-Hernández A, Hafidi ME, Gallardo-Pérez JC, Hernández-Reséndiz I, et al. Mitochondrial free fatty acid β-oxidation supports oxidative phosphorylation and proliferation in cancer cells. Int J Biochem Cell Biol. 2015;65:209–221. doi: 10.1016/j.biocel.2015.06.010 26073129

[11] Bertout JA, Patel SA, Simon MC. The impact of O_2_ availability on human cancer. Nat. Rev. Cancer. 2008;8:967–975. doi: 10.1038/nrc2540 18987634 PMC3140692

[12] Janssen HL, Haustermans KM, Balm AJ, Begg AC. Hypoxia in head and neck cancer: How much, how important? Head Neck. 2005;27:622–638. doi: 10.1002/hed.20223 15952198

[13] Leung E, Cairns RA, Chaudary N, Vellanki RN, Kalliomaki T, Moriyama EH, et al. Metabolic targeting of HIF-dependent glycolysis reduces lactate, increases oxygen consumption and enhances response to high-dose single-fraction radiotherapy in hypoxic solid tumors. BMC Cancer. 2017;17:418. doi: 10.1186/s12885-017-3402-6 28619042 PMC5473006

[14] Sato J, Kitagawa Y, Yamazaki Y, Hata H, Okamoto S, Shiga T, et al. ^18^F-fluoromisonidazole PET uptake is correlated with hypoxia-inducible factor-1α expression in oral squamous cell carcinoma. J Nucl Med. 2013;54:1060–1065. doi: 10.2967/jnumed.112.114355 23699668

[15] Marín-Hernández Á, Gallardo-Pérez JC, Hernández-Reséndiz I, Del Mazo-Monsalvo I, Robledo-Cadena DX, Moreno-Sánchez R, et al. Hypoglycemia enhances epithelial-mesenchymal transition and invasiveness, and restrains the Warburg phenotype, in hypoxic HeLa cell cultures and microspheroids. J Cell Physiol. 2017;232:1346–1359. doi: 10.1002/jcp.25617 27661776

[16] Zhu G, Peng F, Gong W, She L, Wei M, Tan H, et al. Hypoxia promotes migration/invasion and glycolysis in head and neck squamous cell carcinoma via an HIF-1α-MTDH loop. Oncol Rep. 2017;38:2893–2900. 28901527 10.3892/or.2017.5949

[17] Vaupel P, Thews O, Kelleher DK, Konerding MA. O_2_ extraction is a key parameter determining the oxygenation status of malignant tumors and normal tissues. Int J Oncol. 2003;22:795–798. doi: 10.3892/ijo.22.4.795 12632070

[18] Morishima H, Washio J, Kitamura J, Shinohara Y, Takahashi T, Takahashi N: Real-time monitoring system for evaluating the acid-producing activity of oral squamous cell carcinoma cells at different environmental pH. Sci Rep. 2017;7:10092. doi: 10.1038/s41598-017-10893-y 28855722 PMC5577156

[19] Hernández-Reséndiz I, Román-Rosales A, García-Villa E, López-Macay A, Pineda E, Saavedra E, et al. Dual regulation of energy metabolism by p53 in human cervix and breast cancer cells. Biochimica et Biophysica Acta. 2015;3266–78. doi: 10.1016/j.bbamcr.2015.09.033 26434996

[20] Pacheco-Velázquez SC, Robledo-Cadena DX, Hernández-Reséndiz I, Gallardo-Pérez JC, Moreno-Sánchez R, Rodríguez-Enríquez S. Energy Metabolism Drugs Block Triple Negative Breast Metastatic Cancer Cell Phenotype. Mol Pharmaceutics. 2018 Jun 4;15(6):2151–64. doi: 10.1021/acs.molpharmaceut.8b00015 29746779

[21] Mookerjee SA, Nicholls DG, Brand MD. Determining Maximum Glycolytic Capacity Using Extracellular Flux Measurements. PLOS ONE. 2016 Mar 31;11(3):e0152016. doi: 10.1371/journal.pone.0152016 27031845 PMC4816457

[22] Kovačević Z, Morris HP. The role of glutamine in the oxidative metabolism of malignant cells. Cancer Res. 1972;32:326–333. 4400467

[23] Rodríguez-Enríquez S, Pacheco-Velázquez SC, Marín-Hernández Á, Gallardo-Pérez JC, Robledo-Cadena DX, Hernández-Reséndiz I et al., Resveratrol inhibits cancer cell proliferation by impairing oxidative phosphorylation and inducing oxidative stress. Toxicol Appl Pharmacol. 2019;370:65–77. doi: 10.1016/j.taap.2019.03.008 30878505

[24] Cuicui G, Yao S, Fang J, Yajing M, Xiaofei Q., Cancer Stem Cells in Small Cell Lung Cancer Cell Line H446: Higher Dependency on Oxidative Phosphorylation and Mitochondrial Substrate-Level Phosphorylation than Non-Stem Cancer Cells., PLOS ONE., 2016; May 11;11(5):e0154576. doi: 10.1371/journal.pone.0154576 27167619 PMC4863974

[25] Scott DA, Richardson AD, Filipp FV, Knutzen CA, Chiang GG, Ronai ZA, et al. Comparative metabolic flux profiling of melanoma cell lines: Beyond the Warburg effect. J Biol Chem. 2011;286:42626–42634. doi: 10.1074/jbc.M111.282046 21998308 PMC3234981

[26] Bredell MG, Ernst J, El-Kochairi I, Dahlem Y, Ikenberg K, Schumann DM. Current relevance of hypoxia in head and neck cancer. Oncotarget. 2016;7:50781–50804. doi: 10.18632/oncotarget.9549 27434126 PMC5226620

[27] Rofstad EK, Gaustad J-V, Egeland TAM, Mathiesen B, Galappathi K. Tumors exposed to acute cyclic hypoxic stress show enhanced angiogenesis, perfusion and metastatic dissemination. Int J Cancer. 2010;127:1535–1546. doi: 10.1002/ijc.25176 20091868

[28] Akaike T, Nishida M, Fujii S. Regulation of redox signalling by an electrophilic cyclic nucleotide. J Biochem (Tokyo). 2013;153:131–138. doi: 10.1093/jb/mvs145 23248242

[29] Xie L, Xiao K, Whalen EJ, Forrester MT, Freeman RS, Fong G, et al. Oxygen-regulated β2-adrenergic receptor hydroxylation by EGLN3 and ubiquitylation by pVHL. Sci Signal. 2009;2:ra33–ra33. doi: 10.1126/scisignal.2000444 19584355 PMC2788937

